# Biomarkers of balance and gait deficits in *FMR1* premutation carriers: a mini-review

**DOI:** 10.3389/fnagi.2025.1637819

**Published:** 2025-10-06

**Authors:** Leila C. Moore, Nell Maltman, Jonathan S. Lee-Confer, Megan J. Kobel

**Affiliations:** ^1^Department of Speech, Language, and Hearing Sciences, University of Arizona, Tucson, AZ, United States; ^2^Department of Physical Therapy, University of Arizona, Tucson, AZ, United States

**Keywords:** fragile X-associated tremor/ataxia syndrome, postural control, balance, gait, functional mobility, biomarkers

## Abstract

Fragile X-associated tremor/ataxia syndrome (FXTAS) is a neurodegenerative disorder caused by a premutation (PM) of the *FMR1* gene on the X chromosome. FXTAS is characterized by intention tremor, ataxia, and cognitive decline. Age-related cognitive-behavioral and sensorimotor (i.e., balance and gait) abnormalities are also present in PM carriers who do not develop FXTAS. Digital biomarkers of gait and balance have been proposed to be promising markers in characterizing prodromal changes in FXTAS (i.e., preFXTAS) and identifying age-related changes in the *FMR1* phenotype in those who do not develop FXTAS. In this mini-review, gait and balance findings in PM carriers are reviewed to highlight potential future applications. Variability measures of gait and postural sway reveal measurable impairments in individuals with FXTAS, particularly under conditions challenging sensory integration or assessing cognitive-motor interactions. However, there are limited studies quantifying these domains in *FMR1* PM carriers without FXTAS, and there is significant variability in the patient populations assessed (i.e., differing ages, relative lack of information in females) thus restricting conclusions about the progression of balance and gait in FXTAS and possible prodromal markers. Future research should prioritize longitudinal tracking of gait and balance in PM carriers, along with potential cognitive interactions and the characterization of sensory contributions to postural control. This mini-review aims to synthesize current findings on digital balance and gait biomarkers in FMR1 premutation carriers and to explore their potential utility for early FXTAS detection.

## Introduction

The fragile X messenger ribonucleoprotein 1 (*FMR1*) gene is essential for healthy neural development, synaptic plasticity, and nervous system function. In those with a premutation (PM) expansion of CGG repeats on the *FMR1* gene (i.e.,55–200 CGG repeats), approximately 40% of male PM carriers and 16% of females over the age of 50 will develop Fragile X-associated tremor/ataxia syndrome (FXTAS) (Jacquemont et al., 2004). In PM carriers without diagnosed FXTAS, age-related sensorimotor impairments can occur, including increased reports of imbalance (Chonchaiya et al., 2010; Narcisa et al., 2011) and declines in balance and gait performance (Liani et al., 2025; O’Keefe et al., 2015; Timm et al., 2024). However, there is limited information if these balance and gait deficits represent prodromal markers of FXTAS (i.e., preFXTAS) or whether these are part of the broader age-related PM phenotypic spectrum.

Cerebellar degeneration and ataxia are hallmarks of FXTAS (Hashimoto et al., 2011b; Cohen et al., 2006; Wang et al., 2017), and currently, the main methods of gait assessment in clinical settings rely on subjective rating scales. These rating scales are based on examiner qualitative ratings (e.g., Berg Balance Scale) and have consistently identified significant impairment in adults with FXTAS (Timm et al., 2024; O’Keefe et al., 2016; O’Keefe et al., 2021; O’Keefe et al., 2018; O’Keefe et al., 2015). However, there is evidence that clinical assessment scales may underestimate the progression in cerebellar ataxias and severity of gait abnormalities (Schniepp et al., 2016), thus, may be insensitive to preFXTAS or more subtle age-related changes that occur in *FMR1* PM carriers without overt ataxia. In other related degenerative ataxias, quantitative markers of balance and gait are sensitive to pre-ataxic disease stages and disease progression (Milne et al., 2018; Buckley et al., 2018; Ilg et al., 2007; Marquer et al., 2014). Yet there are currently only a relatively small number of studies that have quantified digital biomarkers of gait and balance in PM carriers, both with and without FXTAS. In addition to cerebellar atrophy, *FMR1* PM carriers also display widespread cortical degeneration, thus, there are likely unique features to sensorimotor (i.e., balance and gait) deficits seen in this population in addition to cerebellar ataxic features which warrant further investigation and characterization.

Gait and balance disturbances are reported to be the most disabling features in related disorders (Lowit et al., 2021; Seabury et al., 2023), thus, digital markers of gait and balance may serve as ecologically valid markers of disease progression and may serve to track response to treatment. This mini-review aims to summarize and synthesize findings investigating the role of *FMR1* in sensorimotor function to highlight potential applications of these markers and the need for further research.

### Quantitative balance performance in *FMR1* PM carriers

Multiple studies indicate that male and female PM carriers, both with and without FXTAS, exhibit measurable quiet stance balance (i.e., postural control) deficits (Narcisa et al., 2011; O’Keefe et al., 2015; Aguilar et al., 2008; Allen et al., 2008; Kraan et al., 2013). Balance control is a complex process dependent on sensory input (i.e., visual, vestibular, somatosensory), and generation of an appropriate motor response (Peterka, 2002). As the cerebellum is integral for motor coordination, *FMR1* PM carriers likely have motor contributions to balance deficits. This is supported by findings from O’Keefe et al. (2015) identifying slower automatic postural response times in reaction to unexpected anterior–posterior platform translations on the motor control test in PM carriers both with and without FXTAS which correlated to worse balance performance. However, this is the only study to date which attempted to directly quantify relationships between lower limb motor function and balance.

Typical balance tasks manipulate availability of sensory cues through removing visual cues (e.g., closing eyes) or making somatosensory cues unreliable (e.g., standing on a foam surface), allowing insight into relative sensory contributions for postural control (Halmágyi and Curthoys, 2021; Agrawal et al., 2011; Baloh et al., 1998). Postural sway during balance testing is most often quantified using movements of center-of-pressure (COP) measured with a force plate, and multiple metrics can be quantified describing the displacement, velocity, or frequency of the COP trace, representing unique aspects of postural control (Shah et al., 2024; Maurer and Peterka, 2005). Sway can also be assessed in the mediolateral (ML) and anterior–posterior (AP) planes, rather than across all anatomic planes simultaneously, and past evidence suggests that ML sway may be a more sensitive marker of ataxia (Ilg et al., 2020) and predictor of falls (Maki et al., 1990; Maki et al., 1994).

Prior work suggests that sway velocity is useful in identifying age-related changes in postural control (Prieto et al., 1996) and increases are identified in cerebellar ataxia (Galvão et al., 2022; Van de Warrenburg et al., 2005). Increased overall COP sway velocity (i.e., in both AP and ML planes) is documented in male and female PM carriers with FXTAS when standing on a firm surface, with both eyes open and eyes closed (Narcisa et al., 2011; Aguilar et al., 2008; Allen et al., 2008; Juncos et al., 2011). However, using a similar methodology, male and female PM carriers without FXTAS were not seen to exhibit differences in sway velocity in comparison to age-matched healthy controls (HCs) (Narcisa et al., 2011; Allen et al., 2008), suggesting insensitivity of sway velocity as a prodromal marker of FXTAS or subtle changes occurring in postural control dynamics this population.

Sway displacement magnitude, as quantified by a mechanical sway meter, may also be insensitive to changes in postural sway in PM carriers without FXTAS. For instance, Kraan et al. (2013), did not identify differences between female PM carriers without FXTAS (mean age: 41.32 ± 8.03) relative to age-matched female HCs (mean age: 41.61 ± 8.3) during testing on firm surface, eyes open and eyes closed. However, Birch et al. (2015) identified increased AP and ML sway using similar methodology in male individuals with FXTAS relative to both HCs and PM carriers without FXTAS while no significant differences in sway were identified between HCs to PM carriers without FXTAS. Importantly, groups differed in age (FXTAS: 66.4 years +/−8.1; PM without FXTAS: 47.8 years +/−14.1), which limits comparisons between groups as increased sway in anticipated with increased age (Maki et al., 1994) Further, Birch et al. (2015) only computed the normalized sway factor across all conditions (i.e., eyes open and closed, firm and foam surfaces), rather than assessing quantitative information for each condition independently, limiting insights into specific sensory contributions to postural control in the different populations.

One approach to assessing sensory contributions to balance is the Sensory Organization Test (SOT), which is a standard quantitative balance assessment which systematically removes or degrades reliable visual and/or somatosensory cues. For each test condition, SOT equilibrium scores are provided which quantify maximum AP sway during each condition relative to theoretic age-adjusted maximum sway, in order to identify clinically meaningful decreased postural stability. When using the SOT, O’Keefe et al. (2015) reported significantly higher sway (i.e., decreased equilibrium score) in all conditions in male and female individuals with FXTAS (68.3 years +/− 9.38) relative to HCs (56.9 years +/− 12.31). Similarly, Timm et al. (2024) assessed sway area, root-mean-square distance (RMS), and jerk, representing sway displacement, variability, and velocity in the AP and ML planes, using a single lumbar inertial measurement unit (IMU). Overall, higher sway area, RMS, and jerk were noted for male and female individuals with FXTAS (mean age 68.55 ± 8.31) in comparison to HCs (mean age 64.0 ± 10.45) and PM carriers without FXTAS (mean age 54.94 ± 9.51) during all conditions including eyes-closed and eyes-open on a firm and foam surface. In both Timm et al. (2024) and O’Keefe et al. (2015), differences between individuals with FXTAS to both HCs and PM carriers without FXTAS were largest in conditions with disrupted or removed visual and reliable proprioceptive sensory information, thus, predominantly relying on vestibular sensory information. However, in both studies, individuals with FXTAS were statistically significantly older than controls and PM carriers without FXTAS, partially reflecting later age of onset of FXTAS, but potentially impacting results due to the well-documented impact of age on postural control (Baloh et al., 1995; Baloh et al., 2001).

While significant changes in individuals with FXTAS have been identified in sway velocity, magnitude, and/or variability, changes in sway variability, including RMS, have been identified to be more sensitive to neurodegeneration, and ataxia specifically (Ilg et al., 2020; Ilg et al., 2024). Wang Z et al. (2019) identified increased ML postural sway variability (i.e., COP standard deviation), but not AP sway variability, in male carriers without FXTAS (mean age 61.89 ± 7.40), during eyes open on firm surface testing compared to age-matched male HCs (mean age 57.64 ± 8.92). In *FMR1* PM carriers without FXTAS, Timm et al. (2024) also identified increased RMS and O’Keefe et al. (2015) identified lower equilibrium scores (i.e., increased sway magnitude) only during conditions manipulating visual cues on a firm surface. In both, PM carriers and HCs were well age-matched (i.e., there were no statistically significant differences in age) suggesting changes in processing of visual information relevant for postural control in *FMR1* PM carriers unrelated to age alone.

### Gait and functional mobility in FMR1 PM carriers

FXTAS is in part clinically defined by ataxic gait, however, there is a relative paucity of data assessing gait markers that may help identify preFXTAS or allow differentiation from other movement disorders. Lab-based quantification of gait can be examined using a wide variety of recording technologies including marker-based systems, electronic gait, mats, and body-worn IMUs [for review see (Stephen et al., 2025; Ilg and Timmann, 2013; Ngo et al., 2023)]. In other neurodegenerative disorders and cerebellar ataxias, variability of temporal and spatial measures of gait are strongly related to ataxia severity (Milne et al., 2018; Buckley et al., 2018; Ilg et al., 2007; Marquer et al., 2014). O’Keefe et al. (2016) identified differences in spatiotemporal gait parameters in male and female individuals with FXTAS (mean age 68.78 ± 5.06) relative to HCs (mean age 69.78 ± 5.7) during a 7-meter instrumented Timed Up and Go (i-TUG) at a self-selected pace. Specifically, individuals with FXTAS displayed overall slower gait, spent more time in double-leg support, and higher step variability as quantified using IMUs (O’Keefe et al., 2016). As these groups were well age-matched, this suggests these markers are sensitive to disease state rather than anticipated changes with aging. PM carriers without FXTAS (mean age 67.3 ± 7.35) did not display any significant differences in spatiotemporal gait measures (O’Keefe et al., 2016), suggesting that significant gait ataxia may manifest predominantly after cerebellar neurodegenerative changes accumulate to a critical level. However, a small number of participants were included for PM carriers without FXTAS (*n* = 6) and individuals with FXTAS (*n* = 7), potentially limiting insights into disease progression.

Consistent with other ataxias, gait speed may modulate performance in individuals with FXTAS (Mirelman et al., 2011; Horak et al., 2016; Rennie et al., 2018). Robertson-Dick et al. (2023) observed that during self-selected pace on the 2-min walk test (2MWT), male and female individuals with FXTAS (mean age 69.14 ± 8.12) did not display any significant changes in gait parameters relative to HCs (mean age 62.65 ± 8.52), despite individuals with FXTAS being older than HC participants. However, when performing the 2MWT at a fast-as-possible pace, abnormalities were seen in gait speed, stride velocity, stride velocity variability, and turn duration relative to controls (Robertson-Dick et al., 2023). In contrast, O’Keefe et al. (2021) identified reduced stride length and velocity, swing time, and peak turn velocity and greater double limb support time in individuals with FXTAS (mean age 65.49 ± 8.29) relative to age-matched HCs (mean age 68.87 ± 8.8) for both self-selected and face-paced walking on a 2MWT. Both Robertson-Dick et al. (2023) and O’Keefe et al. (2015) found changes in turn dynamics and movement transitions in individuals with FXTAS. Individuals with FXTAS exhibited longer turn durations during the i-TUG and 2MWT (Chonchaiya et al., 2010; Robertson-Dick et al., 2023) and longer turn-to-sit transitions during the i-TUG (Chonchaiya et al., 2010).

Overall, while significant changes in gait have been consistently seen in individuals with FXTAS, the longitudinal progression of gait performance has not been characterized, limiting the ability to use these biomarkers to track response to future interventions. There is a dearth of studies investigating changes in digital biomarkers in gait performance in PM carriers without a FXTAS diagnosis, limiting insight into the preFXTAS phenotype and broader deficits that may be noted as part of age-related *FMR1* PM progression.

### Cognitive interactions with motor and balance performance

Multiple brain regions including the prefrontal cortex, cerebellum, and cerebellar-cortical pathways subserve both cognitive and motor function (Stuart et al., 2019; Mihara et al., 2008) and demonstrate degeneration in individuals with FXTAS (Brunberg et al., 2002; Hashimoto et al., 2011a; Yang et al., 2013; Adams et al., 2007; Filley et al., 2015). Executive function and information processing deficits are often identified in PM carriers (Jacquemont et al., 2004; Hagerman and Hagerman, 2021) which may reflect dysfunctional frontal networks (Grigsby et al., 2008). As the middle cerebellar peduncles transmit both cognitive and motor information from the frontal lobe to the cerebellum degeneration in this pathway which is common in FXTAS (Famula et al., 2018) may underlie the associations between cognitive decline and motor performance. Several studies point to an interaction between cognitive and sensorimotor function in PM carriers (Timm et al., 2024; O’Keefe et al., 2021; O’Keefe et al., 2018; Kraan et al., 2013) and other neurodegenerative disorders (Wong et al., 2023; Kim and Fraser, 2022). In individuals with FXTAS, slower processing speed, as assessed using the Symbol Digit Modalities test (SDMT), and poorer response inhibition and attention as quantified by the Behavioral Dyscontrol Scale II (BDS-II), was strongly associated with gait speed, slower transitions (e.g., sit-to-stand), and turn velocity. Further, the number of falls was correlated to slower processing speed and poorer response inhibition in individuals with FXTAS (O’Keefe et al., 2018). In *FMR1* carriers without FXTAS, reduced response inhibition (i.e., lower BDS-II score) was correlated to reduced stride velocity and slower transitions for both sit-to-stand and turn-to-sit (O’Keefe et al., 2018). Likewise, reduced working memory using the Letter-Number Sequencing task was associated with slower gait and increased gait variability (Kraan et al., 2014). However, in PM carriers without FXTAS associations to processing speed were not identified (O’Keefe et al., 2018). These findings suggest that the interaction between sensorimotor function and cognition may be more pronounced only after identifiable FXTAS symptoms are present.

Dual-task (DT)interference, in which one performs a motor and cognitive task simultaneously and leading to prefrontal cortex activation (Stuart et al., 2019; Pochon et al., 2001) can lead to decreases in balance and gait performance in PM carriers (O’Keefe et al., 2021; Kraan et al., 2013; Robertson-Dick et al., 2023) and have been proposed to serve as markers of preFXTAS (Timm et al., 2024). Difficulty during DT gait and balance conditions in PM carriers may reflect the known prefrontal cortical and cerebellar neurodegeneration and loss of white matter in frontal-cerebellar circuitry (Hashimoto et al., 2011b; Brunberg et al., 2002; Hashimoto et al., 2011a; Adams et al., 2007). DT interference decreases gait speed and increases double support time in PM carriers, both with and without FXTAS, relative to HCs (O’Keefe et al., 2021; Robertson-Dick et al., 2023; Kraan et al., 2014; Timm et al., 2024). However, some studies have not found a significant increase in DT interference on spatiotemporal gait parameters in both PM carriers with and without FXTAS (O’Keefe et al., 2021; Timm et al., 2024). Sex differences may also play a role in ability to identify DT deficits, as males with FXTAS, but not females, displayed larger impact of concurrent DT on peak turn velocity (O’Keefe et al., 2021).

When assessing balance, during DT conditions, increased AP and ML sway magnitude was seen in female PM carriers without FXTAS with eyes open on firm and foam surfaces relative to HC (Kraan et al., 2013). Additionally, increased RMS sway was identified in male and female PM carriers with and without FXTAS when standing on a firm surface with eyes closed (Timm et al., 2024). Similarly, O’Keeffe et al. (2020) found decreased sway complexity for AP but not ML sway in female PM carriers without FXTAS while performing a DT with eyes open on a firm surface, suggesting a less complex and adaptable sway pattern (O’Keeffe et al., 2019). Increases in sway in DT conditions have been found to be correlated to both CGG length and age (Kraan et al., 2013), suggesting possible age- and genetic- risk profiles for changes in sway. As changes in postural sway were not identified during single-task conditions in both of these studies (Kraan et al., 2013; O’Keeffe et al., 2019), this suggests an inefficiency in cerebellar adaptive mechanisms.

### Genetic and neuroanatomical correlates

The extent of sensorimotor dysfunction in *FMR1* PM carriers appears to be modulated by genetic and neuroanatomical factors. CGG repeat expansion has been most commonly quantified, and overall, CGG repeats linearly correlate with balance outcomes (i.e., higher CGG repeat counts are associated with worse balance performance) (O’Keefe et al., 2015; Birch et al., 2015; O’Keeffe et al., 2019; Wang X. et al., 2019). Overall performance on the SOT (i.e., composite equilibrium score) and reduced performance on conditions with predominant vestibular contributions were predicted by advancing age, male sex, increased CGG repeat size, and reduced X activation of the normal allele in females. In a cerebellar imaging study, Birch et al. (2015) reported that male PM carriers with smaller cerebellar volumes had significantly greater postural sway. Moreover, they identified that this relationship between higher CGG repeat counts and increased sway was mediated by cerebellar volume loss (Birch et al., 2015). CGG repeat length has been identified to be negatively correlated to the adaptability of postural sway, more so under DT conditions, suggesting more rigid, less complex sway patterns (O’Keeffe et al., 2019), hinting at a genotype–phenotype gradient even in preFXTAS or the age-related *FMR1* PM phenotype.

Accumulating evidence links elevated *FMR1* mRNA to motor phenotypes, as those with higher mRNA levels tend to have earlier or more severe FXTAS symptoms (O’Keeffe et al., 2019). Associations between mRNA and gait have yet to be quantified. Only one study to date has assessed associations between *FMR1* mRNA to postural sway and failed to identify a significant association between sway in PM carriers; however, this study examined *FMR1* mRNA levels collapsed across both patients with and without FXTAS, potentially limiting insights into differing phenotypic expression.

### Potential role of vestibular dysfunction

As neural control of motor performance is dependent on sensory input, changes in sensory processing may contribute to balance decline. Particularly, vestibular dysfunction could theoretically play a role in balance deficits as there is higher rate of dizziness reported in female PM carriers relative to HCs (Chonchaiya et al., 2010; Wheeler et al., 2014; Smith et al., 2012). PM carriers with and without FXTAS show the largest changes in postural control performance when visual and somatosensory cues are absent (O’Keefe et al., 2015; Timm et al., 2024), suggesting impaired neural control when using vestibular sensory cues.

Direct clinical evidence of peripheral vestibular system dysfunction in *FMR1* PM carriers or FXTAS has yet to be examined. Further evidence for vestibular involvement in *FMR1* carriers comes from FXTAS mouse models, which show clear deficits in vestibulo-ocular reflex (VOR) function (Foote et al., 2016). In one inducible PM mouse model, the gain of the VOR was significantly reduced which emerged alongside the hallmark cerebellar intranuclear inclusions of FXTAS (Foote et al., 2016). As the middle cerebellar peduncles also transmit sensory information, including vestibular afferent information, to the cerebellum, these findings may suggest inability to use vestibular cues or may suggest overt vestibular dysfunction. However, no studies to date have assessed vestibular function in PM carriers.

A case series by Tak et al. (2024) suggests potential central vestibular involvement due to development of vestibular migraine in three middle-aged female PM carriers. Each of these women experienced new-onset vertigo near menopause, and two reported progressive balance difficulties following vestibular migraine onset which improved or resolved with migraine intervention (Tak et al., 2024). FMRP, the product of the *FMR1* gene, has been suggested to modulate calcitonin gene-related peptide (CGRP) signaling (Mitchell et al., 2023), which plays a significant role in the pathophysiology of migraines (Russo et al., 2014), thus, prevalence of vestibular migraine and related vestibular impairments in PM carriers warrants further investigation.

Overall, while direct vestibular testing in FXTAS is scarce, multiple lines of evidence—from balance tests, rare clinical cases, and animal models—converge to implicate vestibular pathway dysfunction as part of the FXTAS phenotype. Future research using vestibular diagnostics in PM carriers and individuals with FXTAS will be crucial to confirm these speculations.

## Conclusion

Research to date consistently suggests that quantifiable digital biomarkers of gait and balance variability and function can be identified in PM carriers with and without FXTAS. Overall, PM carriers show subtle deficits in balance and cognitive-motor integration under challenging conditions, which may serve as markers of preFXTAS. When sensory contributions to balance performance are considered, vestibular pathways are often implicated due to decreased performance on balance tasks with predominant vestibular contributions (e.g., vision denied, compliant surface). Changes to cognitive function and associated links to postural control and fall history (Buracchio et al., 2011; Blackwood et al., 2016) may further exacerbate motor instability and contribute to an increased risk of falls in FXTAS (O’Keefe et al., 2018; Moser et al., 2021).

Given the relatively small number of studies investigating these sensorimotor changes, the potential role of vestibular function, gait, and balance as biomarkers for preFXTAS or tracking FXTAS disease progression is unknown. Future research should build on these foundational studies with larger longitudinal cohorts, direct vestibular assessments, and quantifying age, sex, and genetic interactions. Identifying reliable digital biomarkers of balance and gait in FMR1 premutation carriers could revolutionize early intervention strategies, particularly if combined with cognitive and sensory profiles. Large-scale longitudinal studies with diverse cohorts are essential to validate these markers and guide personalized treatment approaches.
